# Proteomic Profiling of Human Peripheral Blood Cell Targets of IgG Induced by SARS-CoV-2: Insights into Vaccine Safety

**DOI:** 10.3390/vaccines13070694

**Published:** 2025-06-27

**Authors:** Nicolle Rakanidis Machado, Lais Alves do Nascimento, Beatriz Oliveira Fagundes, João Vitor da Silva Borges, Fabio da Ressureição Sgnotto, Isabella Siuffi Bergamasco, Juliana Ruiz Fernandes, Thalyta Nery Carvalho Pinto, Anna Julia Pietrobon, Gil Benard, Maria Notomi Sato, Jefferson Russo Victor

**Affiliations:** 1 Laboratory of Medical Investigation LIM-56, Division of Dermatology, Medical School, University of São Paulo, São Paulo 05403-000, Brazil; 2 School of Medicine, Santo Amaro University (UNISA), São Paulo 04829-300, Brazil; 3 Post Graduation Program in Health Sciences, Santo Amaro University (UNISA), São Paulo 04829-300, Brazil

**Keywords:** IgG, COVID-19, autoantibodies, SARS-CoV-2, PBMC

## Abstract

Background/Objectives: COVID-19 has been associated with a wide range of immune responses, including the production of autoantibodies, particularly in severe cases. This study investigates the IgG autoantibody responses in patients with varying severities of COVID-19 infection and compares these responses with vaccinated individuals. Methods: We utilized proteomic profiling to analyze autoantibody reactivity against a broad spectrum of proteins expressed in lymphoid and myeloid cell subsets in serum samples from severe and moderate COVID-19 patients, as well as vaccinated individuals who received the inactivated CoronaVac (Sinovac) vaccine. Results: Our findings indicate a marked increase in the diversity and number of IgG autoantibodies targeting intracellular and membrane-associated proteins in severe COVID-19 cases, compared to those with moderate cases of the disease. The autoantibody response in severe cases was found to primarily target proteins involved in immune cell activation, signaling, and differentiation, suggesting potential pathways of immune dysregulation and autoimmunity. In contrast, vaccinated individuals did not exhibit similar autoantibody reactivity, pointing to a more controlled immune response post-vaccination. Notably, no significant autoimmune responses were detected in the vaccinated cohort, suggesting that the inactivated vaccine does not induce autoreactive IgG. These findings align with the established safety profile of COVID-19 vaccines, especially in comparison to the heightened immune dysregulation observed in severe COVID-19 patients. The absence of a significant autoantibody response in vaccinated individuals supports the notion that vaccines, while inducing robust immune activation, do not typically trigger autoimmunity in healthy individuals. Conclusions: Our study underscores the importance of distinguishing between the immune responses triggered by infection and vaccination and highlights the need for the continued monitoring of autoimmune responses in severe COVID-19 cases. Future research should focus on the long-term persistence and clinical relevance of these autoantibodies, particularly in individuals with pre-existing autoimmune conditions or genetic predispositions.

## 1. Introduction

Since the emergence of the COVID-19 pandemic caused by severe acute respiratory syndrome Coronavirus 2 (SARS-CoV-2), more than seven million deaths have been reported globally (Worldometer, 2024) [1]. Although substantial progress has been made in understanding various aspects of SARS-CoV-2 infection—including its epidemiology, pathogenesis, epigenetic mechanisms, clinical management, diagnostic advancements, vaccination strategies, and experimental modeling [2,3,4,5]—comparatively less emphasis has been placed on the virus’s potential to induce autoimmune responses, despite growing evidence suggesting such a link [6,7].

Antibody-mediated autoimmune diseases, which result from complex interactions between genetic susceptibility and environmental factors, affect approximately 10% of the global population and are marked by dysregulated immune activation and systemic inflammation [8,9]. Recent studies, including a comprehensive bibliometric analysis, highlight a growing interest in the link between SARS-CoV-2 infection and the induction of autoantibodies capable of mediating autoimmune responses [10,11,12,13,14].

Several studies have reported the presence of diverse autoantibodies in COVID-19 patients, such as anti-interferon antibodies, cardiovascular and thyroid autoantibodies, autoantibodies linked to rheumatic diseases, and those targeting G-protein-coupled receptors [15]. These immune components may contribute to both acute symptoms and long-term complications of COVID-19, including post-acute sequelae [16]. Emerging data have also pointed to less obvious consequences, such as SARS-CoV-2-associated autoantibodies potentially impairing male reproductive function [17]. However, broader analyses of autoantibody panels during the pandemic have not consistently shown increased seropositivity across various established autoimmune markers, suggesting that many potential targets remain unidentified [18].

A recent investigation involving critically ill COVID-19 patients found that 45% tested positive for antinuclear antibodies, while 15% had detectable levels of antineutrophil cytoplasmic antibodies, indicating a substantial autoimmune component. Nonetheless, results from studies using extracellular antigen profiling—screening thousands of secreted and surface proteins—have yielded conflicting conclusions regarding the pathological role of autoantibodies in COVID-19 [19,20]. These discrepancies likely stem from differences in protein panel composition. Notably, both studies focused exclusively on extracellular and secreted proteins, potentially overlooking intracellular or membrane-associated antigens that may also be targets of autoreactivity.

Further supporting the relevance of autoantibodies, a recent clinical study reported significant symptom improvement in long COVID patients following two cycles of therapeutic apheresis, a nonspecific method of antibody removal [21]. The authors attributed the observed clinical benefits to reduced antibody-mediated autoreactivity, suggesting a broader and possibly underrecognized range of autoantibody targets resulting from SARS-CoV-2 infection.

In a recent investigation by our group, we employed a comprehensive human proteome microarray to evaluate over 23,000 human proteins as potential targets of IgG antibodies induced during moderate and severe COVID-19. This exploratory work initiated the characterization of SARS-CoV-2-induced IgG idiotype interactions across various human cells and organ systems [22].

A recent transcriptomic analysis of peripheral blood mononuclear cells (PBMCs) from COVID-19 patients identified 4843 differentially expressed genes when compared to healthy controls, irrespective of disease severity. This study revealed distinct immune cell signatures, notably implicating neutrophils, monocytes, and CD4^+^ T cells in COVID-19 pathophysiology [23]. Complementarily, a separate proteomic investigation of circulating immune cells uncovered specific cellular phenotypes associated with disease severity, suggesting that peripheral immune cells are actively involved and possibly targeted during the course of infection [24]. Moreover, previous findings have demonstrated that the immune cell composition of peripheral blood closely mirrors that of the pulmonary compartment in COVID-19 patients [25], underscoring the critical importance of investigating immune activation and regulatory mechanisms within peripheral blood cells.

Several peripheral and thymic immune cell types have shown functional modulation in vitro when cultured in the presence of purified IgG derived from various murine or human donors [26,27,28,29,30,31]. These interactions and their modulatory effects suggest a potential in vivo relevance, as demonstrated in patients with atopic diseases [32,33,34,35,36], and have been the subject of ongoing discussion in the literature over the past 15 years [37,38].

Building upon these findings, the present study aims to comprehensively map the proteomic landscape of IgG-mediated interactions with peripheral blood immune cells, including αβ and γδ T cells, B cells, natural killer (NK) cells, myeloid and plasmacytoid dendritic cells, mucosal-associated invariant T (MAIT) cells, neutrophils, basophils, eosinophils, and monocytes. Given the recent global introduction of anti-SARS-CoV-2 vaccines, we also sought to determine whether protein-based vaccination could induce auto-reactive antibodies similar to those observed following natural infection. Our objective is to identify novel candidate proteins that may be targeted by the humoral immune response, particularly in moderate-to-severe stages of SARS-CoV-2 infection. To achieve this, we employed a human proteome microarray comprising 9990 unique human proteins, isoforms, and fragments expressed by immune cells, as annotated by the Human Protein Atlas (www.proteinatlas.org; accessed on 1 May 2025).

## 2. Methods

### 2.1. Sample Collection and Study Cohort

Blood samples were obtained from the Central Laboratory Division of the Clinical Hospital, Faculty of Medicine, University of São Paulo (São Paulo, Brazil). All sera were separated and stored at −20 °C until analysis. Inclusion in the COVID-19 patient cohort required confirmed SARS-CoV-2 infection by a reverse transcription-polymerase chain reaction (RT-PCR). Individuals over 75 years of age and those who tested negative for SARS-CoV-2 were excluded.

The final cohort consisted of 79 COVID-19 patients (39 males, 40 females). Disease severity was classified according to the WHO’s Clinical Management of COVID-19: Living Guideline (18 August 2023). Patients hospitalized with pneumonia who required no oxygen therapy or only low-flow oxygen (via mask or nasal cannula), and who did not meet criteria for severe or critical illness, were categorized as having moderate disease (COVID-Mod; n = 39; mean age: 41.59 ± 5.96 years; 17 males, 22 females). Patients requiring non-invasive ventilation or high-flow oxygen due to severe pneumonia were classified as severe cases (COVID-Sev; n = 40; mean age: 41.78 ± 5.97 years; 23 males, 17 females). Critically ill patients were not included in this study. All COVID-19 samples were collected between May and July 2020, during the initial phase of the pandemic, when vaccines were not yet available to the general population. Consequently, all individuals in the COVID-19 groups were unvaccinated and reflect the natural course of SARS-CoV-2 infection during that period. Detailed clinical data are provided in Supplementary File S1.

As a vaccinated control group, we included 13 healthy individuals (3 males, 10 females; mean age ± SE: 32.5 ± 1.0 years) who had received two doses of the inactivated SARS-CoV-2 vaccine CoronaVac (Sinovac, Pequin, China). Participants were confirmed as COVID-19-negative based on the absence of clinical symptoms throughout the pandemic and seronegativity for anti-SARS-CoV-2 IgM and IgG at the time of the first vaccine dose. Six weeks after the second dose, only individuals with effective neutralizing antibody responses (≥30% inhibition) and no COVID-19 symptoms were retained in the analysis. These were designated as Non-COVID-19 Vaccinated (N-Cov Vac) individuals. Full donor information is available in Supplementary File S2.

A control group of 40 healthy individuals (17 males, 23 females; mean age ± SE: 28.5 ± 2.3 years) whose samples were collected prior to the pandemic (March–July 2019) served as the non-exposed healthy controls (N-exp HC).

### 2.2. Human Proteome Microarray Analysis

We first selected 9990 unique human proteins corresponding to all conserved proteins expressed in the cellular populations included in this study, as identified in the Human Protein Atlas, regardless of expression specificity or intensity. These proteins were subsequently analyzed using our human proteome microarray platform. Proteomic profiling was performed with the HuProt™ Human Proteome Microarray v4.0 (CDI Labs, Mayaguez, Puerto Rico), which contains all selected individual human proteins expressed in Saccharomyces cerevisiae as GST-tagged fusion proteins.

Each protein was spotted in duplicate across 20 blocks, along with control features including fluorescent alignment markers (Rhodamine + IgG647) and assay controls. A comprehensive list of proteins, abbreviations, and related cell types is presented in Supplementary File S3.

Microarrays were washed with PBS (pH 7.4) containing 0.05% Tween 20 (PBST), with each washing step repeated three times for 10 s. Blocking was performed for 30 min using Blocking Buffer (MB-070; Rockland, NY, USA), followed by incubation with serum diluted 1:500 in PBST supplemented with a 10% blocking buffer for 16 h at 4 °C under orbital shaking at 140 rpm.

Detection involved a DyLight 680-labeled goat anti-human IgG (Fc-specific) secondary antibody (0.1 µg/mL), incubated for 45 min at room temperature. Arrays were scanned using an InnoScan 710-IR microarray scanner (10 μm resolution, 680 nm laser, gain 30, low laser power, using the software MAPIX vs4.0.0). Microarray IDs included CDI 9051489–491, 9051493–495, 9051497–501, and 9051695–696.

To assess non-specific backgrounds, arrays were initially incubated with secondary antibody alone. After serum incubation, slides were washed and rescanned. TIFF images (16-bit grayscale) were processed using Mapix 9.1.0 (Innopsys), and data were further analyzed in R (version 4.3.2). Median foreground and local background fluorescence intensities were computed per spot, with duplicates averaged. Background from secondary-only scans was subtracted. Signal-to-background intensity ratios were calculated, and values ≤2 were excluded from downstream analysis. To avoid inflated ratios, a minimum background value of 50 fluorescence units was set.

### 2.3. Identification of IgG Targets and Bioinformatics Analysis

To identify candidate IgG-targeted proteins, we applied a threshold defined as the mean plus three standard deviations (SD) of the non-exposed healthy control (N-exp HC) group, in accordance with recent recommendations [39,40]. Signal intensities from the COVID-19 or vaccinated groups were considered significant if they exceeded this threshold and exhibited a signal ratio greater than 2 relative to the N-exp HC group.

To investigate relevant known and predicted protein–protein interaction networks (PPINs), the identified protein lists were analyzed using the STRING platform (vs12, STRING Consortium, 2024). Analyses included assessments of structural homology, co-expression, Reactome pathways associations, biological processes (Gene Ontology, 2025-03-16), molecular functions (Gene Ontology, 2025-03-16), subcellular localization (COMPARTMENTS), and WikiPathways enrichment.

### 2.4. Statistical Adjustment for Unequal Sample Sizes

To account for the unequal group sizes—particularly the smaller N-Cov Vac group—we applied Welch’s ANOVA for group comparisons involving IgG reactivity profiles, as it does not assume equal variances and is robust to sample size differences. Additionally, non-parametric tests (Kruskal–Wallis followed by Dunn’s multiple comparisons) were employed for independent validation of key findings. All statistical analyses were performed in R (v4.3.2), and significance thresholds were adjusted using the Benjamini–Hochberg method where appropriate.

## 3. Results

### 3.1. COVID-19 Patients Produce IgG Targeting Multiple Peripheral Lymphocyte Populations

All serum samples were initially assessed for reactivity against human proteins expressed in at least one of the selected peripheral lymphocyte subsets, including CD4^+^ T cells, natural regulatory T cells (nTregs), CD8^+^ T cells, γδ T cells, B cells, natural killer (NK) cells, and mucosal-associated invariant T (MAIT) cells. Protein expression data for each cell type were obtained from the Human Protein Atlas (www.proteinatlas.org; accessed on 1 May 2025). For each subset, all proteins expressed in the corresponding cell population were included, regardless of expression specificity or intensity, resulting in a total of 9990 proteins. A comprehensive list of the evaluated and targeted proteins, along with their associated cell-type classifications, is provided in Supplementary File S3. Given the small size of the N-Cov Vac group (n = 13), we conducted additional statistical analyses to confirm the robustness of our results. Welch’s ANOVA was applied to compare mean IgG reactivity scores across groups and was followed by Games–Howell post hoc testing to accommodate variance heterogeneity. These analyses confirmed the lack of significant IgG reactivity in the vaccinated group relative to the COVID-Mod and COVID-Sev groups. Similarly, Kruskal–Wallis tests followed by Dunn’s post hoc corrections reproduced the same group-wise trends, further validating the observed differences. These consistent findings across multiple statistical approaches strengthen the reliability of our conclusions despite the unequal group sizes.

#### 3.1.1. CD4^+^ T Cells

**Figure 1:**
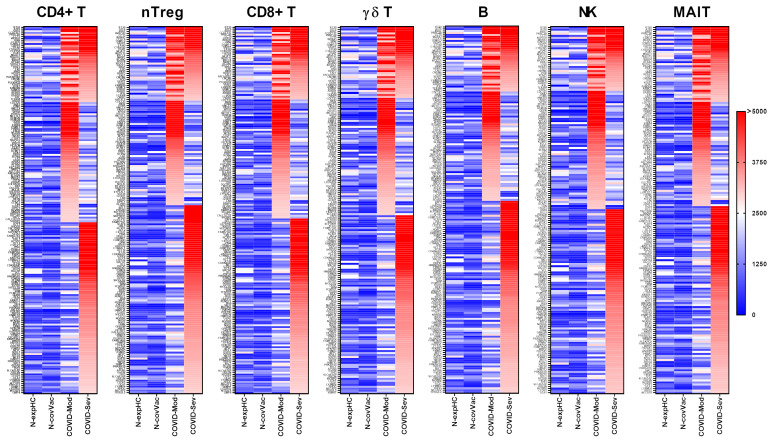
Targeting of Peripheral Lymphocyte Proteins by IgG from different COVID-19 donor groups. Human proteome microarray profiling was performed using serum samples from four groups: 40 healthy individuals unexposed to SARS-CoV-2 (N-exp HC), 13 individuals vaccinated with two doses of the inactivated SARS-CoV-2 vaccine CoronaVac (Sinovac) and testing negative for COVID-19 (N-Cov Vac), 39 patients with moderate COVID-19 (COVID-Mod), and 40 patients with severe COVID-19 (COVID-Sev). Heatmaps show proteins targeted by IgG from each group across various immune cell subsets, including CD4^+^ T cells, regulatory CD4^+^ T cells (nTreg), CD8^+^ T cells, γδT cells, B cells, natural killer (NK) cells, and mucosal-associated invariant T cells (MAIT). Proteins are listed on the left, identified based on reactivity ratios greater than 2 compared to the N-exp HC threshold. The heatmaps are ordered by the intensity of recognition, with proteins most recognized by IgG from the COVID-Sev group at the top, followed by those recognized by both the COVID-Mod and COVID-Sev groups, and proteins exclusively recognized by the COVID-Mod group at the bottom.

IgG from the COVID-Moderate and COVID-Severe groups—either shared or group-specific—intensely recognized 199 proteins expressed in CD4^+^ T cells. Of these, 41 proteins were commonly targeted by IgG from both COVID-infected groups, while 65 proteins were uniquely recognized by the COVID-Moderate group and 93 by the COVID-Severe group. None of these proteins were strongly recognized by IgG from the non-exposed or non-COVID-19-vaccinated control groups (Figure 1).

#### 3.1.2. nTreg Cells

A total of 183 proteins expressed in nTreg cells were intensely recognized by IgG from the COVID-Moderate and COVID-Severe groups. Among these, 37 were recognized by both groups, 52 were specific to the COVID-Moderate group, and 94 to the COVID-Severe group. As with CD4^+^ T cells, none of these proteins were recognized by IgG from the control groups (Figure 1).

#### 3.1.3. CD8^+^ T Cells

We identified 201 proteins expressed in CD8^+^ T cells that were intensely recognized by IgG from the COVID-Moderate and COVID-Severe groups. Of these, 40 were commonly targeted by both groups, 65 were specific to the COVID-Moderate group, and 96 were unique to the COVID-Severe group. No reactivity to these proteins was observed in the control groups (Figure 1).

#### 3.1.4. γδ T Cells

IgG from the COVID-Moderate and COVID-Severe groups also intensely recognized 185 proteins expressed in γδ T cells. This included 36 shared targets, 59 unique to the COVID-Moderate group, and 90 unique to the COVID-Severe group. None were recognized by IgG from the control groups (Figure 1).

#### 3.1.5. B Cells

A total of 185 proteins expressed in B cells were intensely recognized by IgG from the COVID-Moderate and COVID-Severe groups. Of these, 33 were shared, 55 were specific to the COVID-Moderate group, and 97 were specific to the COVID-Severe group. No recognition was observed in control samples (Figure 1).

#### 3.1.6. NK Cells

IgG from COVID-19 patients also intensely recognized 177 proteins expressed in NK cells, with 31 shared across both groups, 57 unique to the COVID-Moderate group, and 89 to the COVID-Severe group. None were targeted by IgG from control individuals (Figure 1).

#### 3.1.7. MAIT Cells

Finally, 180 proteins expressed in MAIT cells were intensely recognized by IgG from the COVID-Moderate and COVID-Severe groups. Among them, 37 were shared, 51 were specific to the COVID-Moderate group, and 92 to the COVID-Severe group. Again, no such reactivity was detected in the control groups (Figure 1).

### 3.2. COVID-19 Patients Produce IgG Targeting Multiple Myeloid-Derived Innate Immune Cell Subsets

All serum samples were initially assessed for reactivity against human proteins expressed in at least one of the evaluated myeloid-derived innate immune cell subsets, including monocytes, myeloid and plasmacytoid dendritic cells (mDCs and pDCs), neutrophils, basophils, and eosinophils. As in the lymphocyte analyses, protein expression data for each cell type were obtained from the Human Protein Atlas (www.proteinatlas.org; accessed on 1 May 2025), with each dataset comprising all proteins expressed in the corresponding cell population, regardless of expression specificity or intensity. A complete list of the evaluated proteins and their associated cell-type categories is provided in Supplementary File S3.

#### 3.2.1. Monocytes

**Figure 2:**
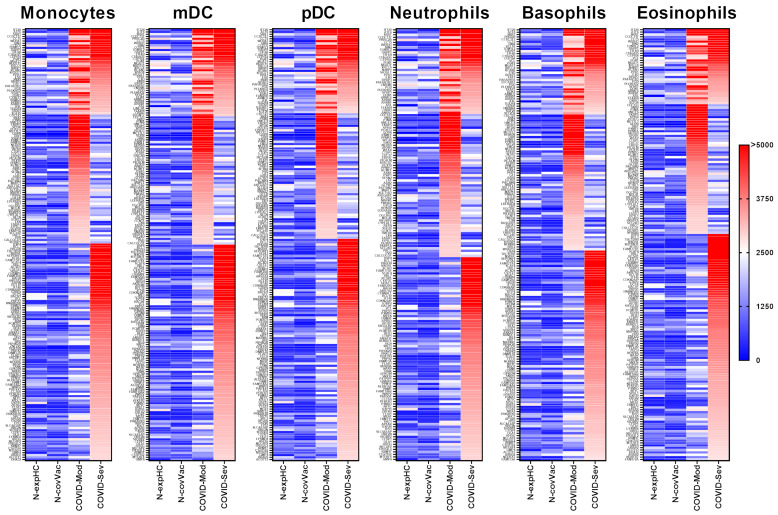
Targeting of Peripheral Myeloid-Derived Immune Cell Proteins by IgG from different COVID-19 donor groups. This figure presents data from human proteome microarray profiling using serum samples from four groups: 40 healthy individuals unexposed to SARS-CoV-2 (N-exp HC), 13 vaccinated individuals (CoronaVac, Sinovac) testing negative for COVID-19 (N-Cov Vac), 39 patients with moderate COVID-19 (COVID-Mod), and 40 patients with severe COVID-19 (COVID-Sev). Heatmaps show proteins targeted by IgG from each group across myeloid-derived innate immune cell subsets, including monocytes, myeloid dendritic cells (mDC), plasmacytoid dendritic cells (pDC), neutrophils, basophils, and eosinophils. Protein targets, listed on the left, were identified based on reactivity ratios greater than 2 compared to the N-exp HC threshold. The heatmaps are organized by recognition intensity, with proteins most recognized by IgG from the COVID-Sev group at the top, followed by shared recognition by COVID-Mod and COVID-Sev groups, and those exclusively recognized by the COVID-Sev group at the bottom.

IgG from both the COVID-Moderate and COVID-Severe groups—either shared or group-specific—intensely recognized 191 proteins expressed in monocytes. Of these, 39 proteins were commonly targeted by IgG from both COVID-infected groups, while 57 were uniquely recognized by the COVID-Moderate group and 96 by the COVID-Severe group. None of these proteins were strongly recognized by IgG from the non-exposed or non-COVID-19-vaccinated control groups (Figure 2).

#### 3.2.2. Myeloid Dendritic Cells (mDCs)

IgG from both COVID-19 groups intensely recognized 192 proteins expressed in mDCs. Among these, 39 proteins were commonly targeted by both groups, 57 were uniquely recognized by the COVID-Moderate group, and 97 by the COVID-Severe group. No strong reactivity was observed in control groups (Figure 2).

#### 3.2.3. Plasmacytoid Dendritic Cells (pDCs)

A total of 189 proteins expressed in pDCs were strongly recognized by IgG from the COVID-19-infected groups. Of these, 37 proteins were commonly targeted, while 55 were unique to the COVID-Moderate group, and 97 to the COVID-Severe group. None were recognized by the control group IgG (Figure 2).

#### 3.2.4. Neutrophils

IgG from the COVID-Moderate and COVID-Severe groups showed strong reactivity to 172 neutrophil-expressed proteins. This included 34 proteins commonly recognized by both groups, 57 unique to the COVID-Moderate group, and 81 to the COVID-Severe group. No such reactivity was observed in control samples (Figure 2).

#### 3.2.5. Basophils

In basophils, 181 proteins were strongly recognized by IgG from the COVID-infected groups. Of these, 36 were common to both groups, 57 were unique to the COVID-Moderate group, and 88 to the COVID-Severe group. None of these proteins elicited strong reactivity in control samples (Figure 2).

#### 3.2.6. Eosinophils

IgG from the COVID-Moderate and COVID-Severe groups intensely recognized 183 proteins expressed in eosinophils. Among them, 32 proteins were commonly recognized, 55 were specific to the COVID-Moderate group, and 96 to the COVID-Severe group. No reactivity was observed in control groups (Figure 2).

### 3.3. Evaluation of Known and Predicted Protein–Protein Interaction Networks (PPINs)

**Figure 3:**
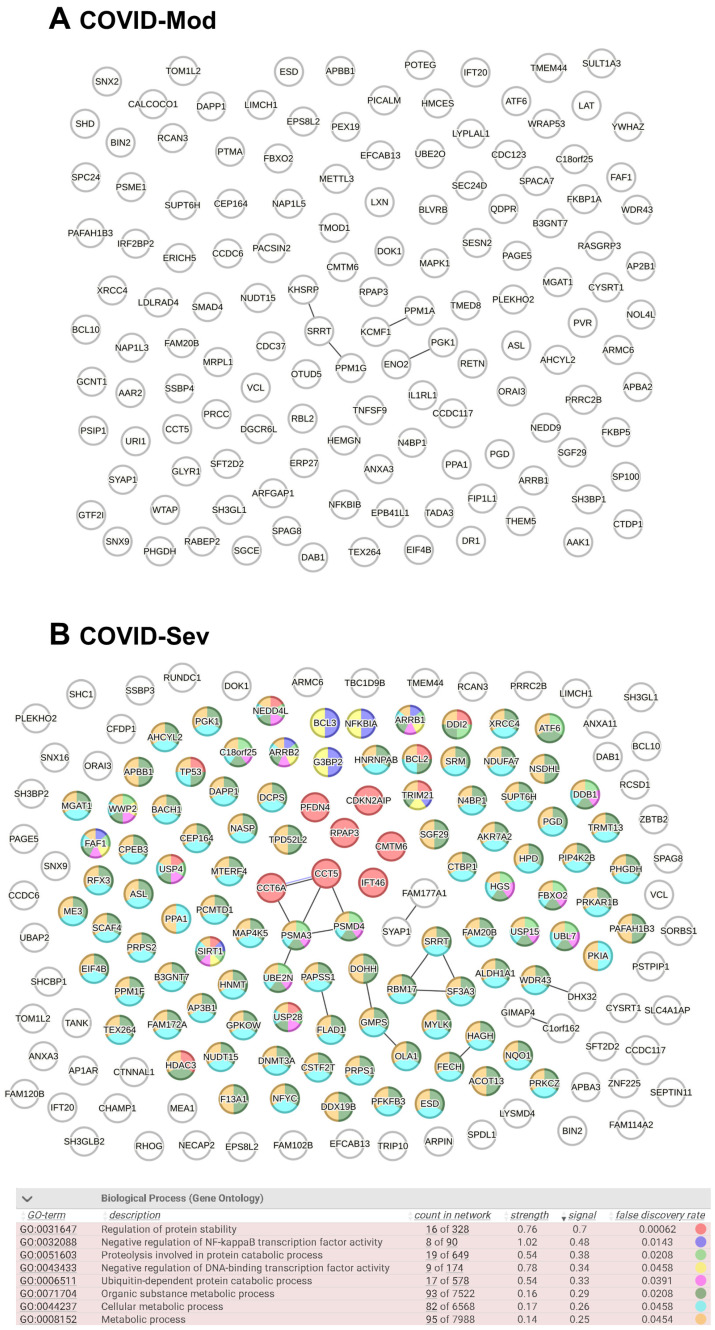
Protein–Protein Interaction Network (PPIN) analysis of COVID-19–induced IgG-targeted proteins. PPIN analyses were performed using the complete lists of proteins targeted by IgG in the COVID-Mod (136 nodes) and COVID-Sev (168 nodes) groups, including proteins targeted by both. Data were analyzed using the STRING platform (STRING Consortium, 2024). Structural homology and co-expression were independently assessed across all identified proteins. In the network diagrams, each node represents a protein target, while edges represent protein–protein associations based on either structural homology (black) or co-expression (purple). Gene Ontology (GO) biological process analysis was conducted to identify significantly enriched pathways (FDR < 0.05). (**A**) No enriched biological processes were identified in the COVID-Mod group. (**B**) In the COVID-Sev group, nodes are color-coded based on their involvement in the most relevant biological processes, as detailed in the accompanying table.

After identifying all cell-associated protein targets in the analyzed samples, we used the STRING platform to evaluate known and predicted protein–protein interactions (PPIs) among these targets. In total, 136 IgG-targeted proteins were identified in the COVID-Mod group and 168 in the COVID-Sev group, including shared targets. Figure 3 displays the PPIN analysis for each group. The COVID-Sev network exhibited a higher density of interactions, with several interconnected modules. Edges in the network represent structural homology (black) or co-expression (purple) between proteins. Notably, Gene Ontology (GO) enrichment analysis identified statistically significant biological processes (FDR < 0.05) exclusively in the COVID-Sev group. These enriched pathways included negative regulation of NF-κB signaling, cellular response to stress, and regulation of apoptosis, as illustrated by the color-coded nodes in panel B.

**Figure 4:**
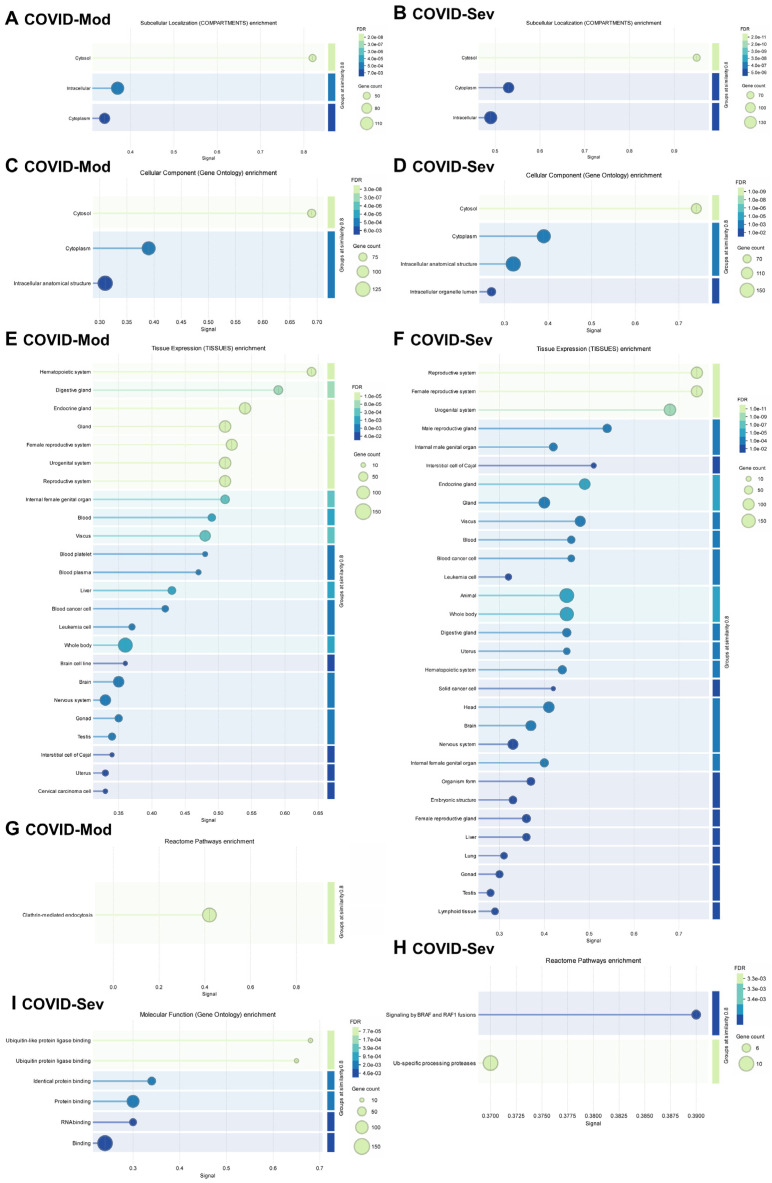
Gene enrichment analysis of COVID-19-induced IgG-targeted proteins. Gene enrichment analyses were performed using the complete lists of proteins targeted by IgG in the COVID-Mod (136 proteins) and COVID-Sev (168 proteins) groups, including shared targets. Statistical significance was determined using a false discovery rate (FDR) threshold of <0.05. Analyses were conducted using the STRING platform (STRING Consortium, 2024). (**A**,**B**) Subcellular localization of enriched proteins was assessed using COMPARTMENTS data. (**C**,**D**) Gene Ontology (GO) enrichment analysis identified significantly enriched cellular components. (**E**,**F**) Tissue-specific localization was determined using TISSUES data. (**G**,**H**) Reactome enrichment analysis identified relevant biological pathways. (**I**) GO enrichment analysis identified significantly enriched molecular functions among the targeted proteins.

Figure 4 presents a detailed gene enrichment analysis of IgG-targeted proteins in both groups. Analysis using the COMPARTMENTS database revealed that most targeted proteins were localized to the cytosol, cytoplasm, and other intracellular compartments (Figure 4A,B). GO Cellular Components analysis confirmed a similar enrichment pattern, highlighting cytosolic, cytoplasmic, and intracellular anatomical structures in both groups (Figure 4C,D).

Tissue-specific enrichment analysis using the TISSUES database showed distinct profiles between the groups (Figure 4E,F). In the COVID-Mod group, IgG targets were more strongly associated with the hematopoietic system, while the COVID-Sev group showed greater enrichment in the reproductive system. Despite these differences, both groups displayed broad tissue diversity in their IgG-targeted proteins, including associations with endocrine tissues, blood, cancer, and leukemia cells. Importantly, the COVID-Sev group uniquely demonstrated enrichment for proteins expressed in lung and lymphoid tissues, which were not observed in the COVID-Mod group.

Reactome pathway analysis revealed further distinctions: in the COVID-Mod group, the most enriched pathway was clathrin-mediated endocytosis, whereas in the COVID-Sev group, key pathways included signaling by BRAF and RAF1 fusions and ubiquitin-specific processing proteases (Figure 4G,H). Finally, GO Molecular Functions analysis highlighted significant enrichment in the COVID-Sev group for protein binding, RNA binding, and ubiquitin–protein ligase binding—suggesting functional convergence on key intracellular regulatory nodes involved in immune modulation. In contrast, no significant molecular function enrichment was observed in the COVID-Mod group (Figure 4I).

## 4. Discussion

The present study demonstrates that SARS-CoV-2 infection induces the widespread production of IgG antibodies targeting intracellular proteins expressed across diverse peripheral immune cell subsets, including both lymphoid and myeloid lineages. These findings reveal a broad and coordinated pattern of autoreactive IgG responses, particularly enriched in patients with severe COVID-19, implicating these immune responses in the immunopathogenesis and clinical trajectory of SARS-CoV-2 infection.

Our data show that sera from COVID-19 patients, especially those with severe disease, contain IgG that intensely recognizes hundreds of intracellular proteins expressed in CD4^+^ and CD8^+^ T cells, B cells, NK cells, MAIT cells, γδ T cells, nTregs, and various innate immune cell subsets, including monocytes, neutrophils, eosinophils, and dendritic cells. The magnitude and diversity of the autoreactive IgG repertoire were substantially higher in the COVID-Severe group than in moderate cases and absent in non-infected, non-vaccinated controls. This observation aligns with accumulating evidence that severe COVID-19 is frequently accompanied by the emergence of autoantibodies targeting various immune and non-immune components [41,42,43,44,45,46,47,48,49,50]. Importantly, none of these cell-associated proteins were targeted by control IgG, strongly suggesting that the COVID-19-induced autoantibody response is disease-specific and not a common feature of physiological immune surveillance. The targets identified include proteins involved in immune cell activation, differentiation, intracellular trafficking, and transcriptional regulation—raising concerns about their potential to disrupt immune homeostasis during and after infection. Our systems-level analysis of IgG-targeted proteins in moderate and severe COVID-19 revealed distinct patterns of protein–protein interactions, subcellular localization, tissue specificity, and functional enrichment that may reflect underlying differences in immune dysregulation and disease pathogenesis.

The higher density of PPINs observed in the COVID-Sev group suggests a more coordinated or intensified humoral immune response to a broader or more functionally integrated set of cellular targets. This finding aligns with reports that severe COVID-19 is often characterized by widespread immune activation and systemic inflammation, which may enhance autoantibody production and epitope spreading [20]. Notably, the enriched PPIN modules identified in the severe group included pathways involved in the negative regulation of NF-κB signaling and apoptosis regulation—two critical processes that are dysregulated in severe SARS-CoV-2 infection [51,52]. Therefore, if these antibodies bind to their targets in vivo, they may interfere with or inhibit the regulatory mechanisms that normally suppress NF-κB activation. This could potentially contribute to sustained or heightened NF-κB activity, aligning with the interpretation provided by the reviewer and supported by the existing literature [53]. The intracellular localization of IgG-targeted proteins, predominantly within the cytosol and cytoplasm, is consistent with recent evidence that SARS-CoV-2 infection induces the production of autoantibodies against intracellular antigens, even though these antigens are typically sequestered from immune surveillance under homeostatic conditions [54]. This phenomenon may reflect the release of intracellular contents during extensive cell death or tissue damage, a hallmark of severe COVID-19 [55], potentially facilitating a break in self-tolerance and the generation of autoreactive IgG. Interestingly, tissue-specific enrichment analysis revealed a marked shift from hematopoietic tissue association in the COVID-Mod group to enrichment in reproductive, pulmonary, and lymphoid tissues in the COVID-Sev group.

The presence of lung- and lymphoid-specific antigens as IgG targets in severe disease may be especially relevant, given that SARS-CoV-2 infection primarily affects respiratory epithelium and is associated with lymphoid tissue destruction [56]. Moreover, reproductive tissue enrichment could reflect systemic immune activation or off-target responses involving tissues that express ACE2 or are susceptible to systemic inflammation [57]. Pathway analysis further underscored divergent immunopathogenic mechanisms. Clathrin-mediated endocytosis, enriched in the COVID-Mod group, is a known mechanism for viral entry and immune receptor trafficking, and may indicate a more localized or regulated immune engagement [58]. In contrast, the COVID-Sev group showed enrichment in BRAF/RAF1 fusion signaling and ubiquitin-specific protease pathways—both of which are implicated in cellular proliferation, stress responses, and inflammation. Notably, the dysregulation of the ubiquitin–proteasome system has been associated with severe COVID-19 outcomes and may contribute to aberrant antigen processing and immune activation [59,60].

Furthermore, the molecular functions enriched exclusively in the COVID-Sev group—including protein binding, RNA binding, and ubiquitin–protein ligase binding—highlight the engagement of regulatory networks involved in post-transcriptional control, proteostasis, and intracellular signaling cascades. These functional nodes may represent converging points of viral interference and host immune modulation, potentially contributing to chronic inflammation, autoimmunity, or immune exhaustion observed in severe and long COVID cases [16,61,62,63,64]. These immunoreactivities may help explain several hallmark clinical manifestations of severe COVID-19. For instance, the pronounced IgG reactivity against intracellular proteins of T and B lymphocytes, NK cells, and MAIT cells may contribute to the profound lymphopenia and functional impairment of these cell subsets frequently observed in critically ill patients [65,66]. Lymphocyte depletion, particularly of CD8^+^ T cells and NK cells, is associated with poor outcomes and may result from direct cytopathic effects, cytokine-mediated apoptosis, or immune-mediated cytotoxicity exacerbated by autoreactive antibodies [67]. Similarly, the enrichment of autoreactive IgG against monocyte and neutrophil proteins in the COVID-Sev group may underlie the myeloid dysregulation and excessive inflammatory cytokine production observed in severe cases, as these cells play key roles in initiating and amplifying inflammatory responses [68]. Furthermore, the identification of lung- and lymphoid-specific targets may help contextualize the pulmonary damage and lymphoid tissue atrophy commonly reported in fatal COVID-19, potentially linking autoantibody-mediated effects with organ-specific pathology [56]. These findings support the hypothesis that SARS-CoV-2-induced autoantibodies are not merely epiphenomena but may actively contribute to disease severity through interference with essential immune regulatory and effector functions.

Beyond lymphoid and myeloid dysfunction, the presence of IgG autoantibodies against intracellular proteins with broad tissue distribution may also help explain some of the extrapulmonary manifestations of COVID-19, particularly neurological and systemic autoimmune complications. Several studies have identified COVID-19-associated autoantibodies targeting components of the nervous system, including antigens involved in axonal transport, synaptic transmission, and myelin maintenance, which have been implicated in post-infectious syndromes such as Guillain–Barré syndrome and autoimmune encephalitis [42,69]. The cross-reactivity of SARS-CoV-2–induced IgG with neuronal proteins may arise through molecular mimicry or bystander activation, particularly in patients experiencing blood–brain barrier disruption during systemic inflammation [70]. Additionally, recent evidence has linked COVID-19 to the development of systemic autoimmune diseases such as systemic lupus erythematosus (SLE) or systemic sclerosis, possibly triggered by sustained autoantibody production, epitope spreading, or persistent immune dysregulation [71,72]. Given that many of the IgG targets identified in our study are involved in transcriptional regulation, cellular stress responses, and protein degradation pathways, it is plausible that their targeting could perturb immune tolerance mechanisms and initiate or exacerbate systemic autoimmunity in genetically predisposed individuals.

From a translational perspective, these findings are relevant to the ongoing investigation of post-acute sequelae of SARS-CoV-2 infection (PASC), commonly referred to as “long COVID.” Several studies have now reported persistent autoreactive signatures months after acute infection, including antibodies targeting immune-related proteins [64,73]. Our results suggest that IgG responses to immune cell-associated proteins may represent part of the immunological “scar” left by COVID-19, potentially contributing to sustained immune dysregulation or autoimmunity in a subset of patients.

In the context of vaccination, these findings underscore the importance of distinguishing infection-induced autoantibody profiles from those generated by immunization. While most studies have shown that COVID-19 vaccines induce protective immunity without broadly stimulating autoreactivity [74,75], isolated reports of immune-mediated adverse events post-vaccination merit further exploration, especially in individuals with predisposing genetic or immunological backgrounds.

This study has several limitations. First, while protein microarrays provide valuable insights into antibody reactivity, they may not fully capture the complexity of in vivo immune responses. Second, cell-type specificity was inferred from publicly available transcriptomic datasets, which may not reflect dynamic expression changes occurring during infection or immune activation. Third, we focused solely on IgG reactivity and did not assess the direct functional effects of these autoantibodies on target cells. Additionally, the cross-sectional design of the study precludes any conclusions regarding the temporal dynamics or long-term clinical implications of the observed antibody responses.

Another limitation concerns the relatively small sample size of vaccinated individuals. This constraint arose because eligible participants were required to meet strict criteria, including strong anti-SARS-CoV-2 IgG response post-vaccination and no symptomatic or serologic evidence of prior infection. As of mid-2021 in Brazil, such individuals were rare due to widespread exposure and varied vaccine uptake. Consequently, identifying suitable participants for the N-Cov Vac group proved extremely challenging.

Importantly, to mitigate the statistical impact of this group size imbalance, we incorporated sensitivity analyses using Welch’s ANOVA and non-parametric tests, which are appropriate for datasets with unequal sample sizes and variance. These complementary analyses reproduced the main findings and provided additional statistical support for our conclusions, reinforcing the robustness of our results.

In conclusion, our study uncovers an extensive network of autoreactive IgG antibodies directed against intracellular proteins across major immune cell populations in COVID-19 patients. These findings highlight a previously underappreciated aspect of SARS-CoV-2 immunopathology, potentially contributing to immune dysfunction during severe disease and its aftermath. Future work should aim to validate these targets in independent cohorts, investigate their temporal dynamics post-infection or vaccination, and determine whether they play active pathogenic roles or represent epiphenomena of immune dysregulation.

## Data Availability

The full datasets used to produce the current study are available from the corresponding author upon reasonable request.

## Supplementary Materials

The following supporting information can be downloaded at: https://www.mdpi.com/article/10.3390/vaccines13070694/s1, Supplementary File S1. COVID-19 DATA. Supplementary File S2. Vax DATA. Supplementary File S3. Evaluated and targeted proteins.
